# Electromyographic correlates of effortful listening in the vestigial auriculomotor system

**DOI:** 10.3389/fnins.2024.1462507

**Published:** 2025-01-31

**Authors:** Andreas Schroeer, Farah I. Corona-Strauss, Ronny Hannemann, Steven A. Hackley, Daniel J. Strauss

**Affiliations:** ^1^Systems Neuroscience and Neurotechnology Unit, Faculty of Medicine, Saarland University & htw saar, Homburg/Saar, Germany; ^2^Center for Digital Neurotechnologies Saar, Homburg/Saar, Germany; ^3^Saarland University, Faculty of Medicine, Homburg/Saar, Germany; ^4^Key Numerics GmbH - Neurocognitive Technologies, Saarbruecken, Germany; ^5^WSAudiology, Erlangen, Germany; ^6^WSAudiology, Lynge, Denmark; ^7^Clinical and Cognitive Neuroscience Laboratory, Department of Psychological Sciences, University of Missouri, Columbia, MO, United States

**Keywords:** effortful listening, electromyography (EMG), objective measures, auricular muscles, superior auricular muscle

## Abstract

Recently, electromyographic (EMG) signals of auricular muscles have been shown to be an indicator of spatial auditory attention in humans, based on a vestigial pinna-orienting system. Because spatial auditory attention in a competing speaker task is closely related to the more generalized concept of attentional effort in listening, the current study investigated the possibility that the EMG activity of auricular muscles could also reflect correlates of effortful listening in general. Twenty participants were recruited. EMG signals from the left and right superior and posterior auricular muscles (SAM, PAM) were recorded while participants attended a target podcast in a competing speaker paradigm. Three different conditions, each more difficult and requiring a higher amount of effortful listening, were generated by varying the number and pitch of distractor streams, as well as the signal-to-noise ratio. All audio streams were either presented from a loudspeaker placed in front of the participants (0°), or in the back (180°). Overall, averaged PAM activity was not affected by different levels of effortful listening, but was significantly larger when stimuli were presented from the back, as opposed to the front. Averaged SAM activity, however, was significantly larger in the most difficult condition, which required the largest amount of effort, compared to the easier conditions, but was not affected by stimulus direction. We interpret the increased SAM activity to be the response of the vestigial pinna–orienting system to an effortful stream segregation task.

## 1 Introduction

Recently, Strauss et al. (2020) demonstrated that electromyographic (EMG) signals of several auricular muscles, specifically the posterior, anterior, superior, and transverse auricular muscles (PAM, AAM, SAM, and TAM), are an indicator of the spatial direction of auditory attention.

This vestigial pinna-orienting system is a so-called “neural fossil” (Hackley, 2015; Strauss et al., 2020), and has a reflexive, stimulus driven component in response to transient auditory stimuli. This component has been observed as transient EMG responses by the PAM, AAM, and TAM, starting approximately 70 ms after rapid-onset auditory stimuli. This part of the vestigial pinna-orienting system does not depend on the participants' voluntary, task-oriented focus, and is therefore referred to as exogenous attention in Strauss et al. (2020), and strongly indicates the direction of the salient auditory stimuli.

The second component of this system is based on deliberately attending an audio stream, while ignoring a competing, but spatially separate stream, and is referred to as endogenous attention. In this case, Strauss et al. (2020) reported sustained activity of the PAM, AAM, and SAM, that was larger on the side of the attended audio stream than on the side of the ignored stream. Furthermore, this effect was enhanced when audio streams were presented outside of the participant's field-of-view (±120°), compared to inside their field-of-view (±30°).

Overall, Strauss et al. (2020) reported differences, especially in the SAM, between purely stimulus driven responses and responses during the active listening task in a challenging condition, that required attentional effort (see Sarter et al., 2006). Listening effort and its relation to different modes of attention and/or cognitive resource limits has been established and analyzed by several models of effortful listening (e.g., Strauss et al., 2010; McGarrigle et al., 2014; Pichora-Fuller et al., 2016; Strauss and Francis, 2017; Herrmann and Johnsrude, 2020). As such, these models are linked to the classic model of attention and effort of Kahneman (1973). For instance, in a 2016 consensus paper, listening effort was defined as “the deliberate allocation of mental resources to overcome obstacles in goal pursuit when carrying out a task, with listening effort applying more specifically when tasks involve listening” (Pichora-Fuller et al., 2016). More recent work also analyzed the interaction of listening effort as defined in this way and affect (see Francis et al., 2016; Herrmann and Johnsrude, 2020).

There is a large body of literature describing many different metrics of listening effort, usually categorized into self-reported, behavioral, and physiological measures. In a literature review by Guijo and Cardoso (2018), the authors found a general lack of consensus about the “best” physiological method to measure listening effort. However, they note that skin conductance appeared to be the most accepted measure at the time. Other measures the authors reported were (in no particular order) pupillometry, EEG (ongoing activity, event-related potentials, and alpha power), EMG (of the frontalis muscles), heart rate (and heart rate variability), skin temperature, and blood pulse rate/amplitude. Other publications investigated the cardiovascular pre-ejection interval (Richter, 2016), functional near-infrared spectroscopy (Wijayasiri et al., 2017), the speech envelope of the EEG (Dimitrijevic et al., 2019), EEG recorded from specialized electrodes at the ear (Ala et al., 2022), facial expressions obtained from video recordings (Venkitakrishnan and Wu, 2023), and electrodermal activity and blood pulse amplitude recorded from wearables (Kondaurova et al., 2024). The last three examples (ear-EEG, video recording, and wearables) should also be highlighted in the context of recording data in a very unobtrusive, ubiquitous manner which is easily integrable outside of traditional laboratory settings.

However, there is also a well described lack of correlation between these measures. For example, Mackersie and Cones (2011) found that skin conductance was correlated with listening effort, but failed to find an effect for the heart rate. Conversely, Seeman and Sims (2015) found the heart rate variability to be a good indicator for listening effort, but the skin conductance was not correlated with perceived listening effort. Miles et al. (2017) found no correlation between pupil responses and alpha band power, but speculate that individually, these measures might be sensitive to different aspects of listening effort. This idea, that different physiological measures are sensitive to different aspects of listening effort has been thoroughly investigated by Alhanbali et al. (2019). The authors simultaneously recorded pupil size, alpha band power, skin conductance, perceived listening effort, and self-reported fatigue during digit in noise recall tasks. The authors reported good test-retest reliability for all measures, except skin conductance and self-reported fatigue, but correlations between measures was reported as weak or nonsignificant. Because measures were poorly correlated with each other, but, reliable during repeated testing, the authors speculate that the lack of correlation is an indicator that these measures are sensitive to different aspects or factors of listening effort. Based on these findings, they grouped measures into four different factors, or underlying dimensions, of listening effort: (1) SNR, hearing level, baseline alpha band power, (2) pupil size, (3) alpha band power during speech processing and retention periods, and (4) perceived listening effort and baseline alpha power.

Shields et al. (2023) performed a literature review, and correlated a large variety of measures related to listening effort to each other. Generally similar to the results reported by Alhanbali et al. (2019), Shields et al. (2023) found statistically significant correlations in only 36.1% of all cases, and if significance was reached, correlation strength was mostly classified as fair (0.3 to 0.6). Therefore, they agree with the idea that different measures are sensitive to different components of listening effort. However, the authors also discuss the influence of the listening task, as well as the time frame when listening effort was measured, which can change the correlation between different measures of listening effort. Overall, the authors highlight that neither the measure, nor the listening task, are freely interchangeable when investigating listening effort, and caution against overgeneralizing results across literature. As a possible way to mitigate this issue, the authors suggest to perform studies less in laboratory environments, and move more toward ecologically valid, real-world scenarios.

For example, Mackersie and Cones (2011) was able to find significant increases of skin conductance and EMG of the frontalis muscles associated with task demand and perceived listening effort. However, they highlight the limitation that their experimental procedure (digits without noise) is relatively simplistic and does not capture the complexity of real-world scenarios. They emphasize the need to perform experiments in more realistic auditory scenes, with complex, or even unpredictable acoustic changes, and the use of acoustically and linguistically complex speech signals.

In a recent review by Keur-Huizinga et al. (2024), the authors found that correlations between different measures of listening effort are often absent or weak. However, they noted that in the majority of the studies they reviewed, auditory tasks were solved with a very high performance (>70%), and those studies often failed to find significant effects on the physiological measures. They argue that in these cases, physiological measures may not be sensitive enough, as such conditions may require an overall low effort level. Therefore, the authors advocate for the inclusion of a broader range of task demand, especially very high (or almost impossible), as well as moderate difficulties.

As a concrete example, Bernarding et al. (2010) and Strauss et al. (2010) recorded EEG during tone and syllable discrimination tasks in noisy, multi-talker environments in young, normal-hearing participants, and found significant increases associated with higher demand conditions and self-reported listening effort. However, in a follow-up study, Bernarding et al. (2013) included not only young, normal hearing participants, but also middle-aged participants with and without moderate hearing loss. To accommodate participants with hearing loss, background noise was removed from the experiment, and found significant differences between task conditions in all middle-aged participants (regardless of hearing status), but not in the young, normal-hearing participants. The authors assume that for young, normal-hearing participants both tasks were equally easy (or effortless) to solve, which is why they were unable to find differences in the EEG. This highlights the importance of using a task that has an appropriate contrast between conditions, which can depend not only on the hearing status of the participants, but also their age, because otherwise physiological measures might be unable to detect effects.

Related to task demand and difficulty, Richter (2016) examined the effect of motivation (or success importance) on a cariovascular measure of listening effort, the pre-ejection period (PEP). They performed an experiment using an auditory discrimination task, which was manipulated in a two-factor design: low and high listening demand, and motivation/success importance, by offering a low and a high monetary reward coupled to the participants performance. The authors found that during the high demand condition, motivation had a very large effect on the PEP (a high motivation led to a larger PEP reactivity). However, during the low demand condition, this difference vanished. The authors discuss the importance of motivation (or success importance) in the context of motivational intensity theory, and highlight the profound impact it can have on physiological measures when attempting to find objective indicators of listening effort. Overall, their results emphasize the importance of a high and constant motivational state of participants during listening effort studies, especially considering that if a task is too difficult to solve, participants might lose motivation, which could be reflected in diminished physiological responses.

Summarizing, there is a well documented interaction between physiological measures (or markers) of listening effort and the listening task, which includes factors such as stimulus material, demand, and motivation. Therefore, important aspects to consider when designing an experiment are the ecological validity of an experiment, the motivation of the participants, and sufficient contrasts between task difficulties, as these are known to influence physiological measures.

In the context of potentially reorienting or shaping the pinna by means of auricular muscle activity, the differentiation between endogenous and exogenous factors for auditory stream selection (see Strauss et al., 2010; Strauss and Francis, 2017) is the main motivation behind the current study. Attempts to alter the physical properties of an auditory stimulus by changing the shape of the pinna and/or ear canal to aid auditory stream segregation seem to be a plausible function of the vestigial pinna–orienting system. Thus we hypothesize that such attempts should be reflected in the EMG of the auricular muscles and could furthermore be enhanced when stimuli are presented from an out-of-view position, as opposed to an in-view position, based on the results from Strauss et al. (2020).

This study will focus on signals obtained from the PAM and SAM for two reasons: First, the effects of sustained auditory attention are strongest at the PAM and SAM (Strauss et al., 2020), i.e., they are known to be significantly modulated by endogenous factors. Second, as the SAM and PAM are the largest auricular muscles (Standring, 2016), and are involved in upward and backward movements (Bérzin and Fortinguerra, 1993), they appear to be the most likely candidates to be involved in an attempt of the vestigial pinna-orienting system to reorient or reshape the pinna during effortful listening. However, as the PAM and SAM displayed a considerable degree of lateralization when target and distractor streams were spatially separated (Strauss et al., 2020), we will remove the spatial separation between target and distractor as a possible confounding factor. Furthermore, the difficulty of the task will be manipulated by two factors, both of which are along the so-called “demand dimension” (as opposed to motivation) in a model for effortful listening proposed by Pichora-Fuller et al. (2016): the fundamental frequency differences between target and distractor, as well as the signal-to-noise ratio (SNR) between target and distractor.

## 2 Materials and methods

### 2.1 Participants

Twenty adult native German speaking participants without any known neurological or cognitive deficits were recruited for this study (12 male, 8 female). They were, on average, 28 ± 4 years old and normal-hearing (pure tone audiograms using test frequencies from 125 Hz to 8 kHz were below 25 dB HL). The experiment was explained to every participant in detail before they signed a consent form.

### 2.2 Experimental setup

**Figure 1:**
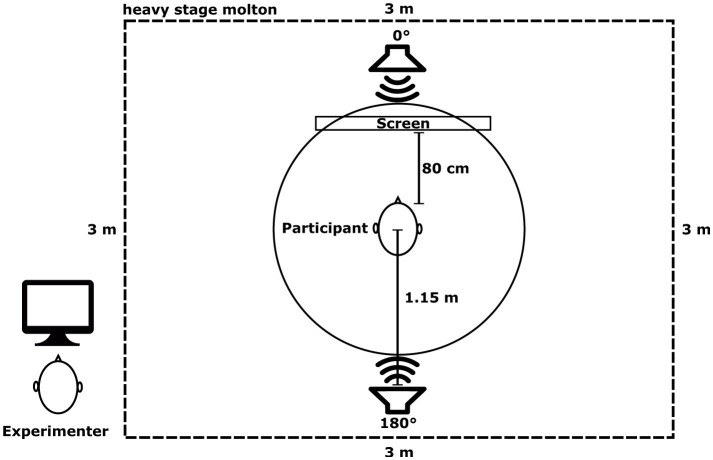
Experimental setup indicating the positions of the loudspeakers around the participant inside the 3 × 3 × 3 m cubicle made of heavy stage molton. Note that the experimenter remained outside of the cubicle.

Participants were seated in a chair in the center of a 3 × 3 × 3 m cubicle made of heavy stage molton (900 $\frac{g}{m^{2}}$) to reduce sound reflections (a T20 reverberation time of 102 ms was achieved). In order to avoid head movements during the experiment, the participants heads were placed on a chin-rest. Two active loudspeakers (KH120A, Neumann, Germany) were placed in front (0°) and behind (180°) the participants, at a distance of 1.15 meters and at head level (see Figure 1). A screen with a fixation cross was placed 80 cm away from the participants head, below the loudspeaker placed at 0°, therefore not blocking the loudspeaker. The setup was calibrated using a Brüel & Kjær Type 2250 Sound Level Meter at an ambient noise level of 25.7 dB LAeq (A-weighted equivalent continuous sound level). During calibration, the Sound Level Meter was placed at the position of the participant's head, facing upward, and dB LAeq values for every audio file (targets and distractors) were measured separately for their respective duration. A dedicated PC controlled the loudspeakers via an external USB sound interface (Scarlett 18i20, Focusrite Plc., UK), generating audio and trigger signals at 44.1 kHz.

### 2.3 Stimuli and tasks

Three different podcasts/audiobooks were used as target and distractor stimuli. For the target streams, segments of an audiobook, spoken by a female speaker, who briefly discusses a variety of topics (approximately 1 min per topic) were used. Two different radio podcasts, one spoken by a male speaker, one by a female speaker (similar to the target) were utilized as distractors. Because their runtime was shorter than the experiment, we systematically shifted the starting points of the distractor podcasts so no trial had the same “background” noise. Audio stimuli were chosen for several reasons. Both target and distractor stimuli were required to be professionally recorded, i.e., a high audio quality, as well as dialect-free, clear and consistent, single speaker. Furthermore, they were supposed to only consist of speech, i.e., no non-speech sound effects, as these might attract a lot of auditory attention. The audiobook for the target stimulus was chosen because it satisfied the aforementioned criteria, and additionally, its content consisted of a variety of topics that we thought were generally interesting and non-contentious, which ensured that participants were easily motivated to attend the target stimuli throughout the experiment. For the distractor streams, we specifically selected two podcasts where one had a speaker with a very different voice, and one where the speaker had similar voice compared to the target stimulus (in addition to the quality requirements). Pauses during speech, defined as the 100 ms long moving average of the rectified digital waveform having an amplitude of less than 0.001 a.u., in all stimuli were removed. These parameters were determined experimentally by listening to the audio files afterwards to validate that no words were cut off. This procedure was done in order to increase the overall difficulty, as the removal of speech pauses increased the information density and prevented random unmasking effects.

During the experiment, participants were instructed to attend to the target podcast, while ignoring the distractor podcasts. Target and distractors were always presented from the same loudspeaker (both were presented from 0°, or both were presented from 180°), i.e., there were no spatial cues segregating target and distractor streams.

Three different conditions, each designed to be more difficult and require a larger amount of effort, were designed. Similar to Koelewijn et al. (2015) and McGarrigle et al. (2021), acoustic properties of the target stream remained constant throughout all three conditions, i.e., the target stream was always presented at 50 dB LAeq by the same female speaker (same voice pitch). However, we altered several factors of the competing distractors to emulate a more realistic, ecologically valid setting: Imagine a person sitting in, for example an almost empty restaurant, attempting to listen to a person. In such a setting, it would be expected that there would be only one or a few other people talking, and their overall loudness would be relatively low, hence being almost effortless. However, if the hypothetical restaurant would become more busy, a larger amount, and an increasing variety of speakers would be present, generating a higher ambient noise level, which would increase the effort required to listen to the person talk, while ignoring all other distractors. To somewhat emulate such a scenario, with increased difficulty, the distractors became louder, increased in number, and became more varied (different speaker/pitch). In the condition designed to be the least effortful (easy condition), the distractor was 10 dB softer then the target podcast (40 dB LAeq, +10 dB SNR). Additionally, the distractor was a male speaker, meaning a high voice pitch difference between (female) target and (male) distractor. For the medium condition, an additional, female distractor was added with a voice pitch similar to the target podcast. Furthermore, both distractors combined were only 2 dB softer than the target (45 dB LAeq each, +2 dB SNR). For the difficult, and therefore most effortful condition, the SNR was further lowered to -2 dB by increasing the distractor intensities to 49 dB LAeq each. Table 1 summarizes the stimulus intensities of the three aforementioned LE conditions. The specific SNR values were determined based on feedback obtained during preliminary testing, while the basic manipulation of number and pitch of distractors was fixed. The easy condition was supposed to be rated almost effortless, and the difficult condition very effortful, but still solvable, to prevent participants from giving up on the task. Once these two SNRs were found, the SNR of the medium condition was then determined by finding an SNR that participants perceived as having a noticable contrast between both, easy and difficult condition.

**Table 1:** Stimulus intensities and SNR values for the three conditions, each designed to make it more effortful to attend the target speaker, which remained constant throughout the conditions at 50 dB LAeq, while the number and intensity of distractors systematically increased.

**Condition**	**Target [dB LAeq] (female speaker)**	**Distractor 1 [dB LAeq] (male speaker)**	**Distractor 2 [dB LAeq] (female speaker)**	**Sum Distractors [dB LAeq]**	**Sum All [dB LAeq]**	**SNR [dB]**
Easy	50	40	N/A	40	50.4	+10
Medium	50	45	45	48	52.1	+2
Difficult	50	49	49	52	54.1	–2

It should be noted that we specifically avoided any spatial separation between target and distractor streams because strong non-spatial features, such as voice pitch differences, are known to interact with spatial cues (Bonacci et al., 2020), and could therefore be a confounding factor when recording auricular muscle activity. For example, spatial separation might have no influence during the easy condition, where strong pitch-based differences are present (Bonacci et al., 2020), but could significantly lower the required listening effort during the medium/difficult condition, where strong non-spatial features are not readily available (Fintor et al., 2022).

Considering two stimulus directions and three effortful listening conditions, a total of six combinations were possible. For each combination, two trials of 5 min and 10 seconds each (12 trials in total) were recorded. In the first 5 seconds of each trial, only the target speaker was active, giving the participant the opportunity to solely focus on the target stream. During the next 5 seconds, the distractor(s) linearly faded in. Only the remaining 5 min of the trial, during which the distractor steams were at full intensity, were used for data analysis. The presentation order of the six combinations was randomized and balanced across participants, but the trials of each combination were always the same.

After each trial, participants rated their subjectively perceived listening effort on a 7-point scale (from effortless to extreme) and gave an approximate number of how often they lost the target stream during the trial (up to 10). Then, to ensure that participants had not given up during the experiment, they were instructed to recall the topics discussed in the corresponding target podcast trial (on average 4 topics per trial), as well as answer open, content-related questions. For example, a topic participants were expected to recall was: “How does a chameleon change its color?” and a corresponding content-related question would be: “What are the special skin cells of the two skin layers of a chameleon made of?” It should be noted that perceived listening effort scores and the number of target streams lost were always asked before the topic recall and content-related questions in order to avoid any bias based on the participants' impression of how well they were able recall the topics and answer the associated questions. Furthermore, participants were encouraged to take small breaks between trials to minimize fatigue.

### 2.4 EMG data acquisition

**Figure 2:**
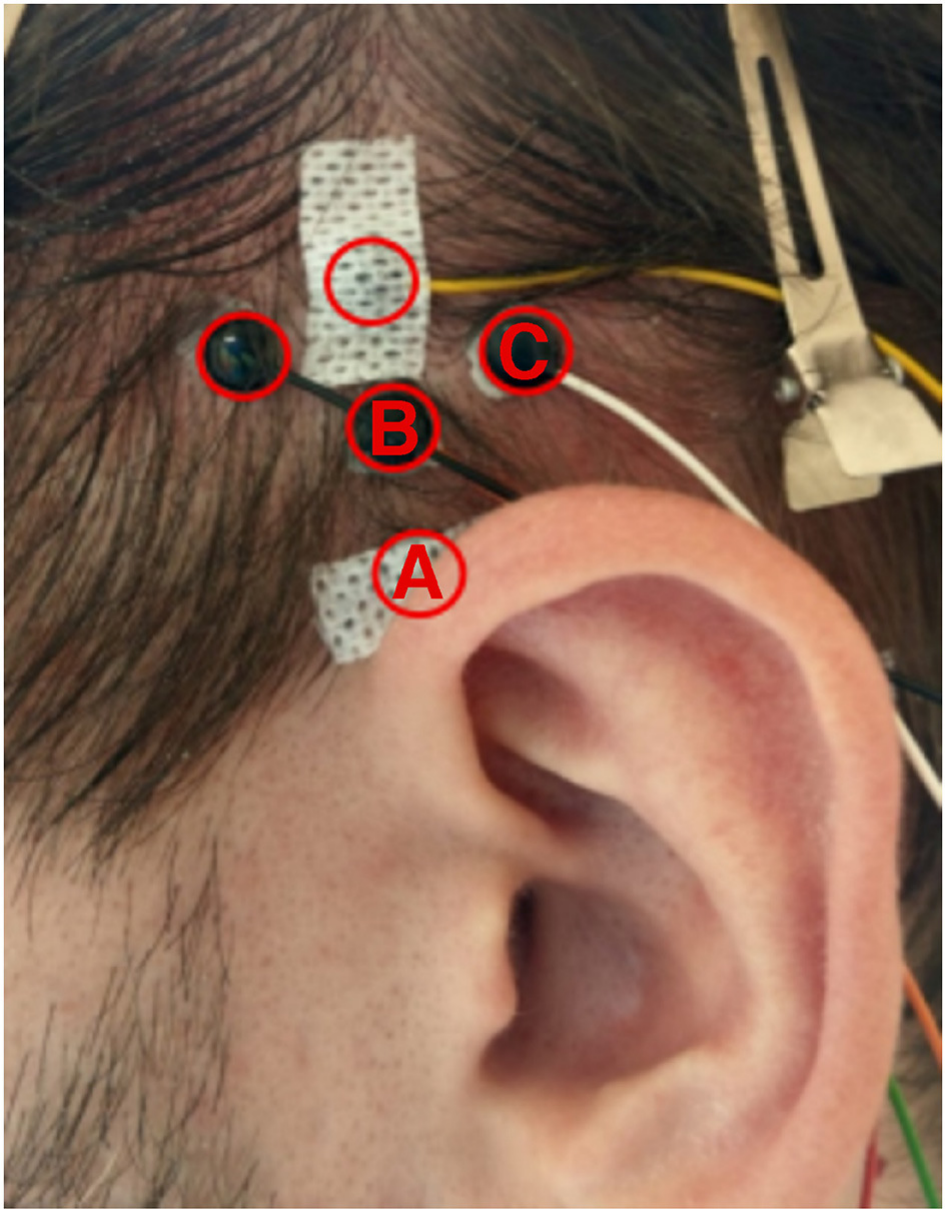
Positions of the five electrodes used to cover the SAM.

Passive Ag/AgCl electrodes were used to record EMG signals from the left and right superior auricular and postauricular muscles (SAM, PAM), as well as the masseter muscles (M. masseter). The PAM, the second largest auricular muscle, is located directly behind the ear, approximately on the height of the ear canal, connects the mastoid bone to the posterior surface of the pinna (Standring, 2016), and is approximately 2.8 cm long and 0.9 cm wide (Millard et al., 2022). Electrode placement for this muscle was identical to Strauss et al. (2020): one electrode was placed where the PAM connects to the pinna, and the another centrally on the PAM (the PAM can easily be made visible by gently pulling on a participants' pinna, see Figure 6 in Schroeer et al., 2023). The SAM, which is the largest auricular muscle, is fan-shaped, with the narrower part attached at the cranial surface of the pinna, and the wider part the galea aponeurotica (Standring, 2016; Chon et al., 2021). Importantly, the SAM is reported to greatly vary in size: the central length ranges from 2.5 to 6 cm, and the width from 4 to 6.5 cm (Chon et al., 2021), and, as opposed to the PAM, cannot be made visible on the surface to ensure proper electrode placement. This motivated us initially to apply five electrodes in a diamond-shaped pattern in order to cover the complete area where the SAM should be located (see Figure 2). Comparing the electrode placements in Figure 2 to the photograph of the SAM in Chon et al. (2021) (Figure 2), the electrode positions labeled B and C in Figure 2 are most likely to be positioned on the SAM. The M. masseter was recorded by placing one electrode on each side, slightly below the temporomandibular joint (the point which strongly protrudes during teeth clenching). One concern was that participants could, due to the long use of the chin-rest, move their jaw or clench their teeth during the experiments, activating the temporal muscle which could be picked up by the electrodes placed on the SAM. Because both the M. masseter and temporalis muscle (M. temporalis) are involved in these movements, signals recorded from the M. masseter will later on be used to remove potentials artifacts. All electrodes were initially referenced against the ground electrode, which was placed at the upper forehead (Fpz). All signals were recorded at 4,800 Hz using a commercially available, direct current (dc)-coupled, biosignal amplifier (g.USBamp, g.tec, Austria).

### 2.5 Signal processing

Signal processing and statistical analyses were performed using Matlab 2020a (Mathworks, USA), IBM SPSS Statistics 28 (IBM Corp, USA), and R 4.2.1 (R Core Team, 2022). Raw EMG signals were initially re-referenced to bipolar signals. For the PAM, both electrodes on one side were used for re-referencing, resulting in one bipolar PAM signal per side. For the SAM, the average of electrodes B and C were referenced against electrode A. Signals from the left and right electrodes placed on the M. masseter were used to calculate one bipolar M. masseter signal. All signals were 10–500 Hz bandpass filtered (3rd order butterworth) and a 50 Hz IIR comb filter was applied (all filters were implemented as zero-phase filters). Signals were then segmented into 1 second long, non-overlapping segments.

Next, artifact rejection was performed, independently for every trial and participant, based on two metrics. The first metric is based on the M. masseter signal. If the mean absolute value of any (1 second) segment exceeded 10 μV, then the corresponding segments of the auricular signals were flagged as artifacts and discarded from further analysis. The threshold of 10 μV was determined experimentally, based on data where every participant was first instructed to sit in a relaxed state, and then to clench their teeth on command. Across the participants, 10 μV appeared to be a value that reasonably separated deliberate teeth clenching from spontaneous baseline activity. The second metric was based on the auricular signals themselves. The energy of every 1 second long segment was calculated, and any segment that deviated by more than two standard deviations from the mean energy of the corresponding trial was removed from further analysis. Table 2 summarizes the artifact rates based on all participants and trials.

**Table 2:** Averaged artifact rates and standard deviations based on all 20 participants. Values for for PAM and SAM were obtained by calculating the energy of non-overlapping 1 second long segments, and rejecting segments that deviated by more than two standard deviations from the corresponding mean value. For the M. Masseter, non-overlapping 1 second long segments whose mean absolute value exceeded 10 μ*V* were rejected.

**Muscle**	**Artifact rate [%]**
SAM	5.35 ± 3.39
PAM	5.6 ± 3.58
M. Masseter	2.8 ± 4.04

In Figure 6 of Strauss et al. (2020), the authors showed data that could suggest a decrease of auricular EMG activity with time, possibly indicating fatiguing or adaption effects. However, during analysis, while plotting time-resolved EMG data, we unexpectedly observed what appeared to be a trend that the contrast between the effortful listening conditions increased approximately 2.5 min into the trials (halfway through the trial), and diminished in the last few seconds. Because of this unexpected observation, we decided to split the data into first and second half (2.5 min seconds each), and add this as a *post-hoc* factor for analysis. Finally, for every direction (0° and 180°) and all three effortful listening conditions, the mean energy from all valid 1 second long segments was calculated. These averaged values were then z-normalized, independently for every participant and muscle (left/right PAM, SAM, M. Masseter), and subjected to statistical analysis by means of a four-factor repeated measures ANOVA: 3 effortful listening conditions (easy, medium, difficult) × 2 stimulus directions (0° and 180°) × 2 time frames (first and second half) × 2 muscles (left and right - PAM and SAM only). Critical alpha values for statistics were set at α = 0.05. When Bonferroni-corrections for multiple comparisons were applied, the corresponding p-values were increased, i.e., alpha values remained at α = 0.05. Furthermore, perceived listening effort scores and the number of how often participants lost the target stream were z-normalized, while results from topic recall and content questions were converted to percent correct prior to statistical analysis.

## 3 Results

**Figure 3:**
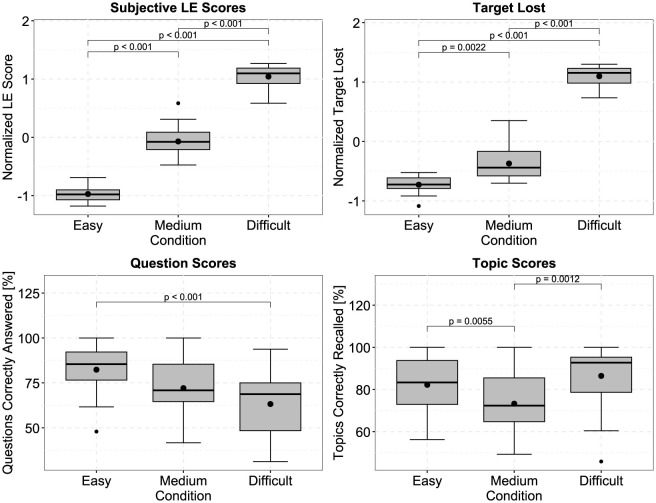
Averaged values of the normalized perceived listening effort (LE) and target lost ratings, as well as percentages of correctly answered questions and topic recall scores. Both, LE scores and target lost values significantly increase when the paradigms become more effortful. The differences between easy and medium are much larger when considering the target lost, than the LE scores. Question and topic scores are primarily used to indicate that participants attempted to solve all paradigms, and did not give up or disengage during the difficult condition. *P*-values were obtained using Bonferroni corrected paired t-tests (*df* = 19). Black dots outside of the boxplots indicate outliers.

Figure 3 displays the averaged subjective ratings (perceived listening effort and how often the participants lost the target stream) per effortful listening condition after z-normalization, as well as the scores of the correctly answered questions and topics recalled. For the subjective listening effort rating and target lost metric, the boxplots show a clear increase with task difficulty. Repeated measures ANOVAs with the effortful listening condition and stimulus direction as factors indicated significant effects of the effortful listening condition for the subjective listening effort rating [$F_{\left(2,38\right)}=336.332,p<2\cdot 10^{-16},\;\eta_{p}^{2}=0.947$] and how often participants lost the target stream [$F_{\left(2,38\right)}=303.929,p=2.1\cdot 10^{-15},\;\eta_{p}^{2}=0.941$]. Pairwise t-tests (*df* = 19, Bonferroni corrected) show that, for self-reported listening effort and number of target streams lost, each effortful listening condition significantly differs from another. In Figure 3, it can be observed that the averaged ratings of the perceived listening effort almost form a straight line (values for easy, medium, and difficult are: –0.972, –0.0704, and 1.0424),i.e, there is an almost equal spacing between the difficulties (0.9016 and 1.1128), possibly indicating a comparable increase of perceived listening effort. For averaged values for the number of target streams lost, however, we found that the increase from medium to difficult was much larger than from easy to medium (values for easy, medium, and difficult are: –0.7269, –0.3707, and 1.0977, with differences being 0.3562 and 1.4684). There were no significant effects of stimulus direction or interactions. Regarding the question scores, significant main effects were observed for both the effortful listening condition and stimulus direction [effortful listening condition: $F_{\left(2,38\right)}=6.696,p=0.003,\;\eta_{p}^{2}=0.261$, stimulus direction: $F_{\left(1,19\right)}=11.715,p=0.003,\;\eta_{p}^{2}=0.381$]. Participants made significantly more errors in the difficult condition, compared to the easy condition (mean percent correct scores for easy, medium, and difficult: 82.35%, 72.08%, 63.23%). As for recalling the topics, there was only a significant main effect of the effortful listening condition [$F_{\left(2,38\right)}=11.637,p<0.001,\;\eta_{p}^{2}=0.38$]. Significantly fewer topics were recalled during the medium condition compared to either the easy or difficult condition (mean values for easy, medium and difficult: 82.21%, 73.26%, 86.44%). There were no significant interactions.

**Figure 4:**
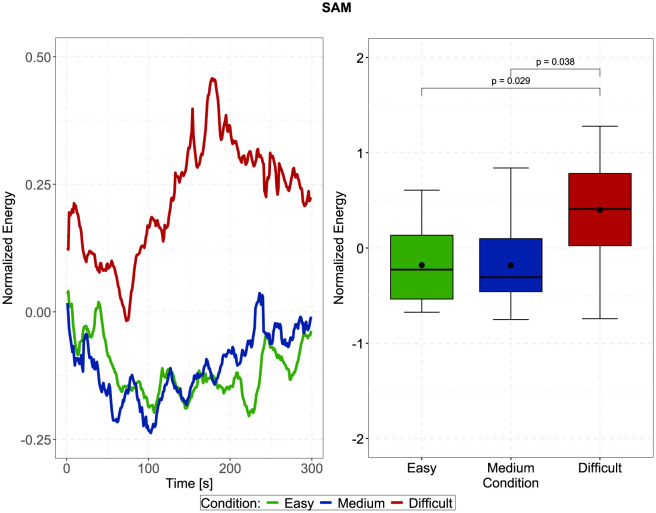
**Left**: Time-resolved normalized activity of the superior auricular muscle (SAM) depending on the three effortful listening conditions. There appears to be a trend that the contrast between the difficult and easy/medium conditions increases with time, and diminishes in the last few seconds. **Right**: Averaged and normalized SAM activity according to the effortful listening conditions. SAM activity was significantly larger during the difficult condition than during the easy and medium conditions. *P*-values were obtained using Bonferroni corrected paired t-tests (*df* = 19).

**Figure 5:**
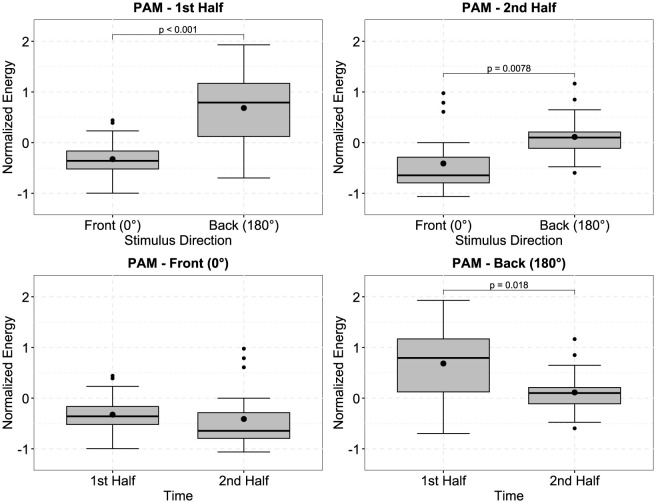
Boxplots of the normalized energy of the posterior auricular muscle (PAM), depending on the stimulus direction and time. PAM activity was significantly larger when stimuli were presented from the loudspeaker located behind the participants, than from the loudspeaker in the front (**top left**). For data from the second halves of the trials, the same effect was observed (**top right**). When comparing PAM activity from the first halves to the second halves of the trials, there was no significant difference when stimuli were presented from the front (**bottom left**), but activity was significantly larger during the first half, when stimuli were presented from the back (**bottom right**). *P*-values were obtained using Bonferroni corrected paired t-tests (*df* = 19). Black dots outside of the boxplots indicate outliers.

In Figure 4, the left plots show the normalized and time-resolved plots of the SAM, averaged across all trials and participants, in 10-second steps, to generate a more smoothed curve. Visually, there appears to be an increased contrast between the difficult and easy/medium conditions in the second half of the trials, i.e., after approximately 150 seconds. However, the repeated measures ANOVA did not indicate a significant main effect of time, side (difference between left and right SAM), or stimulus direction. In fact, the only significant effect was the main effect of the effortful listening condition [$F_{\left(2,38\right)}=6.523,p=0.004,\;\eta_{p}^{2}=0.256$]. Pairwise Bonferroni-corrected *t*-tests furthermore indicated that SAM activity during the difficult condition was significantly larger than during the easy and medium conditions, which is furthermore displayed in the right plot of Figure 4 [difficult compared to easy: *t*_(19)_ = −2.872, *p* = 0.029, estimated difference: −0.58, 95%-CI: (−1.111, −0.05); difficult compared to medium: *t*_(19)_ = −2.754, *p* = 0.038, estimated difference: −0.583, 95%-CI: (−1.139, −0.027)]. Considering data from the PAM, we observed significant main effects of stimulus direction [$F_{\left(1,19\right)}=21.813,p<0.001,\;\eta_{p}^{2}=0.534$], time [$F_{\left(1,19\right)}=4.467,p=0.048,\;\eta_{p}^{2}=0.19$], as well as a significant interaction between these factors [$F_{\left(1,19\right)}=4.6,p=0.045,\;\eta_{p}^{2}=0.195$]. *Post-hoc* contrasts (displayed in Figure 5) indicated that for both, first and second half of the trials, z-normalized PAM activity was larger when stimuli were presented from the back, than from the front: First half: *t*_(19)_ = −4.587, *p* < 0.001, estimated difference: −1.008, 95%-CI: (−1.469, −0.548); Second half: *t*_(19)_ = −2.971, *p* = 0.008, estimated difference: −0.523, 95%-CI: (−0.892, −0.155). However, when stimuli were presented from the back (180°), PAM activity in the first half was significantly larger than in the second half of the trials [*t*_(19)_ = 2.58, *p* = 0.018, estimated difference: 0.571, 95%-CI: (0.108, 1.034)], but not when stimuli were presented from the front (0°), which explains the interaction effect.

Signals obtained from the M. masseter did not show any significant main effects or interactions [effortful listening condition: $F_{\left(2,38\right)}=0.41,p=0.666,\;\eta_{p}^{2}=0.021$; stimulus direction: $F_{\left(1,19\right)}=0.012,p=0.891,\;\eta_{p}^{2}=0.001$; time frame: $F_{\left(1,19\right)}=0.055,p=0.818,\;\eta_{p}^{2}=0.003$]; *p*-values for interactions were all above 0.593.

**Figure 6:**
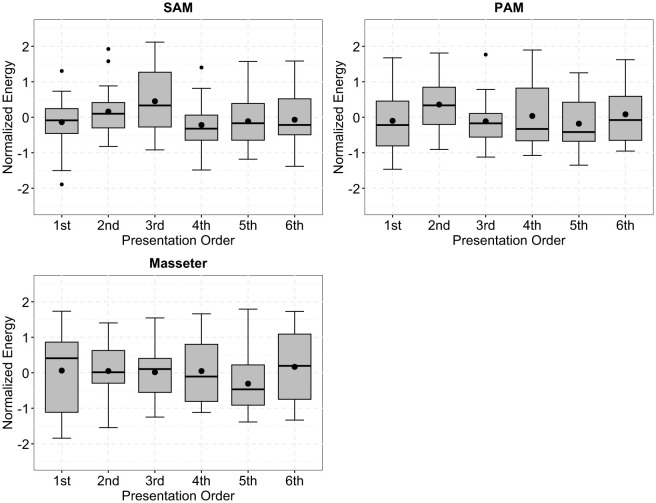
Boxplots of the normalized energy of the superior auricular muscle (SAM), posterior auricular muscle (PAM), and M. masseter, arranged to be in presentation order for every participant during the experiment. There were no significant differences associated with the presentation order, indicating that there were no fatiguing or habitation effects. Black dots outside of the boxplots indicate outliers.

Figure 6 shows data from the SAM, PAM, and M. masseter arranged according to presentation order for every participant. One-way repeated-measures ANOVAs did not indicate a significant effect of presentation order for SAM, PAM, or M. masseter [SAM: $F_{\left(5,95\right)}=1.762,p=0.128,\;\eta_{p}^{2}=0.085$; PAM: $F_{\left(5,95\right)}=0.998,p=0.424,\;\eta_{p}^{2}=0.05$; M. masseter: $F_{\left(5,95\right)}=0.508,p=0.77,\;\eta_{p}^{2}=0.026$].

## 4 Discussion

Sustained activity of auricular muscles has been shown to reflect the spatial direction of auditory attention (Strauss et al., 2020), using a vestigial pinna-orienting system (Hackley, 2015). Based on these findings, we designed an experiment to determine if this vestigial system could also be active during more generalized scenarios involving effortful listening. We generated conditions that require several distinct levels of effortful listening (as indicated in the perceived listening effort scores), based on the number and pitch of distractors (the demand dimension, see Pichora-Fuller et al., 2016) while purposefully not spatially separating target and distractor streams to avoid lateralization effects [as reported in Strauss et al. (2020)]. At the same time, we included two levels of stimulus direction (presentation of all streams from either 0° or 180°), because auricular responses were reported to be larger when stimuli were presented from outside the participants' field-of-view (Strauss et al., 2020).

We found that signals from both left and right SAMs generally displayed significantly more activity during the difficult and more effortful condition, compared to the easy and medium condition. Easy and medium conditions were, however, not significantly different. A surprising finding, even though it was not significant, was the potential trend of an increased contrast between the difficult and easy/medium condition after approximately 150 seconds. It is surprising insofar as the sustained SAM activity in response to spatial attention reported in Strauss et al. (2020) displayed a declining trend or remained stable over time, which might be attributed to the detrimental effect of prolonged time on task as described in Sarter et al. (2006). In the design phase of the study, we specifically decided to record shorter and more trials (2 × 5 minutes instead of one 10 minute long trial). This was done because we initially speculated that the EMG activity could display a downwards trend [similar to Strauss et al. (2020)]. Additionally, we wanted to avoid participants disengaging from the task due to fatigue or demotivation/disengagement, which plays a pivotal role in listening effort research (Herrmann and Johnsrude, 2020; Francis and Love, 2020), and could have an adverse effect on the manipulation of listening effort by introducing changes along the motivation dimension (Pichora-Fuller et al., 2016). Therefore, the implication for future studies regarding effortful listening using auricular muscles is that if trials are too short, they may fail to capture this effect. Conversely, it would be interesting to study the time course of the SAM beyond the 5 minute mark in order to assess how long this effect lasts, and if we actually captured the “maximum” contrast between effortful listening conditions, or if there is another increase (see the time-resolved plot in Figure 4).

Mackersie and Cones (2011) recorded EMG signal from the frontalis muscles, which, like the auricular muscles, are innervated by the 7th cranial nerve (Ottaiano et al., 2023), during three different levels of task difficulty, and found a significant increase from medium to high difficulty. Given the shared neural structures between auricular and facial muscles, the question arises if the increased auricular muscle activity observed in the current study is independent of or related to the increased frontalis activity reported in Mackersie and Cones (2011). Raising of the eyebrows, which is the purpose of the frontalis muscles, has been documented to substantially increase PAM activity, only surpassed in magnitude by smiling, laughing and deliberate ear movements (Lipede et al., 2023). Because the current study did not find increased PAM activity associated with effortful listening, we could speculate that increased SAM activity is independent of the frontalis activity reported in Mackersie and Cones (2011). However, because Mackersie and Cones (2011) utilized different stimuli and paradigms than the present study, directly comparing results between studies should be done with caution. Instead, future studies should probably record the frontalis muscles alongside auricular muscles to investigate a potential co-activation. Another facial muscle, the corrugator supercilii, which is also innervated by the 7th cranial nerve, was recorded by Francis et al. (2021) during challenging listening conditions, but the authors were unable to observe any significant effect between two different levels of listening effort. They speculate that this could in part be due to the low affective valence of the stimuli used (i.e., slightly negative to neutral emotional stimuli), which the corrugator supercilii is an indicator of. Considering potential co-activation between the corrugator supercilii and the SAM, the function of corrugator supercilii is to draw the eyebrows down, which can result in a moderate increase in SAM activity using surface electrodes (Rüschenschmidt et al., 2022), and no or only a slight increase using invasive electrodes (Bérzin and Fortinguerra, 1993; Rüschenschmidt et al., 2022). So while there is some evidence for co-activation between these muscles during facial movements, to our knowledge, there is currently no evidence to suggest an effect of affective valence of auditory stimuli on the SAM response. Nevertheless, because the stimulus material used in the current study across effortful listening conditions was from the same audiobook and speaker, the affective valence of the stimuli should be mostly constant throughout the experiment and should not be a confounding factor.

Furthermore, Francis et al. (2021) reported an effect of SNR on self-rated effort, but not on physiological measures, which includes the corrugator supercilii. The authors suggest that within a certain stimulation range, sound level related effects are negligible on physiological responses. This interpretation could be supported with the data of the present study: the sound level differences between the low and medium LE condition are large enough for significant differences in self-reported perceived listening effort, but not for the physiological response (in this case, the SAM). For the difficult condition, on the other hand, the sound level of the distractors might be high enough (and therefore, the SNR low enough) to generate responses of both, self-reported perceived listening effort, as well as physiological signals.

The coupling between the visual system and auricular muscles has been long documented (see, for example, Wilson, 1908; Patuzzi and O'Beirne, 1999; Liugan et al., 2018). While movement of the eyes can be effectively controlled during experiments (Strauss et al., 2020; Schroeer et al., 2023), controlling facial muscles, such as the frontalis and corrugator supercilii is not as straightforward. Even though results of facial muscles as a measure of effortful listening appear to be somewhat mixed (Mackersie and Cones, 2011; Francis et al., 2021), we do believe that measuring facial muscles alongside auricular muscles during effortful listening conditions would be a worthwhile addition, as this could (a) reveal the degree of association (or confirm the independence) of facial and auricular muscles in listening conditions, and (b) act as a supplementary signal, which could be used to improve the differentiation between effortful listening conditions.

Comparing the collected SAM data to the self-reported perceived listening effort scale, the SAM does not capture the difference in the reported listening effort ratings between the easy and medium conditions, which is comparable to the difference between the medium and difficult condition. Perhaps this difference may be explained by a recent review by Shields et al. (2023), which analyzed the correlation between different measures of listening effort and concluded that correlation between effort questionnaires and physiological measures were mostly poor to fair, and only 28.8% were significantly correlated. Another possible explanation might be a bias introduced by the participants. Brännström et al. (2018) performed experiments without noise, and in a +10 dB SNR babble noise. While they were unable to find significant effects on behavioral measures, self-reported listening effort was significantly larger in the +10 dB SNR condition, which corresponds to the SNR of the easy condition in the current study, which we designed to be almost effortless. We should therefore consider the option that participants might have noticed the very low noise in Brännström et al. (2018), realized that this condition is “supposed” to be more effortful, and reported perceived listening effort accordingly. So, instead of concluding that behavioral measures are not sensitive enough, self-reported measures might be biased because participants are aware of the “ground truth” of the conditions. We cannot exclude that something similar happened in the current study, when looking at the perceived listening effort scores. As mentioned in the results, the averaged listening effort ratings are almost equally spaced, and non-normalized differences from easy to medium to difficult are approximately 1.3 and 1.6, i.e., only 1–2 points higher, which would indicate a sequential rating which could be solely based on the participants realization of the task difficulty or presumed “ground truth.”

A different self-reported measure (how often participants lost the target stream), appears to capture the results of the SAM more closely, namely a much smaller difference between the easy and medium condition. Considering the possible bias in the self-reported listening effort scores, the target lost metric could be more reliable, as the mapping between the “suspected ground truth” and the target lost metric is less straightforward, and therefore potentially less biased, which can be supported by the observation that the contrast between conditions is more similar to the recorded auricular EMG. Additionally, in Pichora-Fuller et al. (2016), the authors developed a three dimensional model, in which effort is a nonlinear function of demand and motivation. Assuming a constant level of motivation, the results from both the SAM and the target lost metric could easily fit onto such a curve: even if the demand between the easy, medium and difficult conditions (as quantified by the self-reported perceived listening effort questionnaire) would be evenly spaced, the proposed nonlinear relationship between demand and effort could result in a negligible difference between easy and medium conditions and substantial increase in effort during the difficult condition [see the computational model of the demanded and exerted effort relation in Schneider et al. (2019)]. However, we did not ask *how long* participants lost the stream, which could potentially differentiate between the easy and medium conditions.

On the other hand, instructing the participants to keep track of the number and duration of how often they lost the target stream would be an additional task and might severely distract from their primary objective.

Nevertheless, there is a growing consensus that measures of listening effort (or effortful listening) depend on different underlying dimensions, are not interchangeable, and depend on a complex interaction between external and internal factors, such as fatigue, motivation, and attention (McGarrigle et al., 2014; Strauss and Francis, 2017; Alhanbali et al., 2019; Herrmann and Johnsrude, 2020; Francis and Love, 2020). Therefore, we could interpret the recorded SAM data in the context of losing the target stream, which, on average, participants did once in the easy, twice in the medium, and six times in the difficult condition. We could speculate this to be the vestigial pinna-orienting system's attempt to change the spectral properties of the pinna or the ear canal. The evolutionary purpose of this could be to lower the external/perceptual listening effort, as opposed to the internal/cognitive listening effort (Strauss and Francis, 2017; Francis and Love, 2020), and therefore aid to “locate” the target stream.

Strauss et al. (2020) has shown that transient and sustained involuntary activity of auricular muscles can lead to visible movements or deformations of the pinna shape. If such a movement is large enough, as in many mammals such as cats and dogs, this would impact the head-related transfer function (HRTF, see Stitt and Katz, 2021). Whether or not movements of the auricular muscles can affect the shape of the pinna in humans to such a large degree that a utilizable change is generated would require a dedicated, future study that includes appropriate video recordings in a calibrated recording setup and specialized computer vision algorithms as suggested by Strauss et al. (2020). If attention-driven auricular movements are purely vestigial in our own species, clues as to their original function might be discerned from other primates. Directly stimulating the 7th nerve branch to SAM in an anesthetized macaque (see the Supplementary material of Waller et al., 2008) showed that maximum contraction yields an upward, essentially rigid, translation of the pinna relative to the ear canal. The pinna as a whole does not appreciably rotate or deform. A consequent shift in distance of the upper and lower walls of the concha (which is of special importance in determining the HRTF, see Stitt and Katz, 2021) might generate a simple, predictable change in the spectral properties of the proximal stimulus while maximizing the aperture of the ear canal. By contrast, isolated contraction of the PAM or AAM homologs yields more complex, multidimensional movements in which the tragus can occlude the ear canal. As an example of exaggerated changes in the shape of the pinnae in humans, Shirota et al. (2019) used a head mounted device to mechanically apply pressure to differentially alter the shape of both pinnae. The authors were able to significantly alter the perceived location of an acoustic object in the frontal plane. Related to this, Stevenson-Hoare et al. (2022) bypassed the pinnae by inserting extension tubes into the ear canals, which might be the exaggerated example of maximally retracting the pinnae, minimizing its filtering properties and maximizing the accessibility of the ear canal. Without the extension tubes (a normal pinna), perceived sound localization was significantly better in the frontal hemifield, compared to the rear hemifield. Insertion of the tubes, however, completely removed this difference, i.e., the presence of the pinna significantly contributed to the perception of sound in space. These two studies obviously utilized completely unnatural and unrealistic alterations to the pinna in humans. However, as both studies were able to quantify distinct and different changes in perception, they could represent the upper limit of the influence of pinna movements in humans. An interesting intermediate step would be a study which exclusively includes participants who are able to voluntarily move their ears. As those movements are within the natural capabilities in humans, corresponding perceptual changes would represent a closer approximation of the capabilities of the vestigial pinna-orienting system. Apart from voluntary movements, Strauss et al. (2020) has also provided evidence of sustained increased auricular EMG activity during an active listening task, which lead to a visible (without video magnification) upwards movement of both pinnae for the entirety of the audio stimulation (5 min, see video 3 in Strauss et al., 2020). While this was only reported in one participant, and should therefore not be overgeneralized, it does demonstrate that the auricular system in humans can cause a longstanding, visible deformation of the ear canal and translation of the pinnae as a whole during a listening task. However, when discussing the potential effect of pinna movements in humans, it is important to consider that the presence of head movements has a profound influence on the perception of sounds and is much more readily available. In the current study, we specifically avoided head movements by using a chin-rest, but also by avoiding lateralized stimuli, as these could incentivize head movements to reduce task difficulty. On the other hand, animal studies have demonstrated that head and ear movement do not have to be in competition but can work together in a precise manner. Tollin et al. (2009) has shown that when cats were prompted to rotate their head toward an acoustic stimulus, two types of ear movements can be observed. Initially, with a very short latency, the pinna was oriented toward the sound. Next, as the head started to turn toward the sound, a slow pinna movement was observed, which compensated the head movement to keep the pinna “pointed” toward the sound. While it is of course difficult to compare the behavior observed in animals with highly mobile pinnae to humans, Friauf and Herbert (1985) found a similar topographical organization of the facial motor nucleus (which innervates the auricular muscles) in rats and bats, which could suggest a similar organization in all mammals.

The behavioral responses (question and topic recall scores) show a less clear picture than the self-reported listening effort and target lost metric. While the question scores do display a decline with increased listening effort, only the difference between the low and high LE condition reached statistical significance. Topic recall scores even show significantly lower scores in the medium condition compared to both easy and difficult. However, we should mention again that both scores were designed to check general participant compliance, i.e., whether participants stopped solving the task due to, for example, boredom (if scores in the easy condition were very low), or if they gave up (low scores in the difficult condition). Both cases could have effects on the physiological measures (Herrmann and Johnsrude, 2020). Note that trials had a varying number of topics (2–7) and associated questions (1–4) and were fixed to a corresponding effortful listening condition. For example, a topic about animal behavior contained a question that many participants failed to answer and was always part of the medium LE condition. Interpreting these scores is therefore difficult, because there may be some systematic bias present. This could also be an explanation for the significant effect of stimulus direction on the question scores. It is possible that the questions associated with the stimulus material presented form 180° are simply significantly easier. Nevertheless, both scores were, on average, above 63% (questions) and 73% (topics).We believe this indicates that participants consistently attempted to solve the paradigm and retained a certain level of motivation, especially since the content-related questions were almost entirely open questions, i.e., participants giving up would be reflected in a score of almost 0%.

During *post-hoc* analysis, we observed that the activity of both PAM muscles was significantly affected by the direction of the stimuli (0° vs. 180°), and not by the different effortful listening conditions. Specifically, PAM activity when attending audio streams from the back was significantly larger than attending the front.

While PAM activity was also larger when participants attended stimuli from the back in Strauss et al. (2020), their experiments focused specifically on spatial auditory attention, i.e., target and distractor streams were spatially separated. Furthermore, the loudspeakers in Strauss et al. (2020) were not placed directly in front of or behind the participants, but off-center at ±30° and ±120°. Combining the current results with Strauss et al. (2020), we can conclude that the PAM is generally more responsive to audio streams that are outside of the participants' field-of-view. This could lead us to hypothesize that if the eye gaze cannot shift toward a stimulus, the vestigial pinna–orienting may activate the PAM to enhance the participant's ability to focus on these sounds.

The primary potential confounding factor in this study was cross-talk from the M. temporalis, which is situated extremely close to the SAM. Specifically, we were concerned that participants might begin to grind their teeth during the experiment, due to their positioning on the chin-rest becoming uncomfortable over time, or as a general response to stress. The masseter muscle, which works in conjunction with the M. temporalis during mastication, should provide a good proxy signal to assess possible cross-talk between the SAM and M. temporalis. Because analysis of the M. masseter revealed no significant effect of the effortful listening conditions or stimulus direction, it seems unlikely that the signals recorded from the SAM are the result of cross-talk from the M. temporalis. Another concern could be increased activity from facial muscles during the difficult condition. However, the bipolar electrode configurations have good spatial selectivity, good enough to record different motor unit action potentials from the SAM and PAM [see Figure 5 in Schroeer et al. (2024)], which are closer to each other than muscles involved in facial movements. If EMG cross-talk from facial muscles would be present, we would have expected it, at least to some degree, to be also present at the PAM. Additionally, Strauss et al. (2020) did record the left and right zygomaticus and frontalis muscles, and did not find any correlations with results obtained from electrodes placed at several auricular muscles, including PAM and SAM. Similarly, Rüschenschmidt et al. (2022) compared results from needle and surface electrodes at several auricular muscles, and found that surface EMG signals originated from the auricular muscles, and not from larger neighbouring muscles.

Furthermore, Bérzin and Fortinguerra (1993) recorded EMG signals from the auricular muscles while participants performed tasks such as forcefully opening or closing their eyes, making vertical wrinkles on the forehead, lowering the eyebrows, and blinking the eyes, but were not able to identify increased activity at the SAM. Rüschenschmidt et al. (2022) conducted a similar study and reported no to moderate increases at the SAM when participants were instructed to draw their eyebrows, depending on the electrode configuration (needle, single channel surface or multi-channel surface electrodes). However, it should also be mentioned that such forced, exaggerated facial movements are expected to be considerably larger than subconscious facial movements that might be associated with effortful listening.

There are serval limiting factors that should be emphasized. Participants formed a relatively small and homogeneous group, i.e., young and normal-hearing, which has been shown to have an effect on physiological measures of listening effort (Bernarding et al., 2013; Alhanbali et al., 2019). While Strauss et al. (2020) did not find significant differences of auricular muscle activity in relation to participant age, spatial auditory attention and effortful listening have different modulation effects on the auricular muscles, as PAM activity can be significantly enhanced during spatial auditory attention (Strauss et al., 2020), but not during effortful listening. Especially in the context of potentially utilizing auricular muscles as a tool to evaluate auditory processing algorithms (e.g., in hearing aids) to reduce listening effort, inclusion of participants with hearing loss and other age groups is a necessary step.

Furthermore, the anatomical variability of the SAM, and therefore, electrode placement is an issue that has to be addressed in the future. For more fundamental, controlled future studied, utilization of needle electrodes (similar to Rüschenschmidt et al., 2022) could be useful, as needle electrodes are also more robust against potential muscle cross-talk, due to their higher spatial selectivity. On the other hand, high density electrodes grids could be employed to systematically explore the distribution of the electrical activity of the SAM, even though this would be restricted to a smaller subset of participants without hair at the SAM. As Rüschenschmidt et al. (2022) has described voluntary movements which maximally activate the SAM, decomposition algorithms (which require a large amount of densely placed electrodes) could be used to obtain detailed motor unit activity maps and inform future studies on an ideal surface electrode placement.

While we believe the current experiment to be pointed toward an ecologically valid scenario, key factors, such as spatially distributed noise, and potentially moving sound sources (which offer important information for source segregation, see Cho and Kidd, 2022) should be included in future studies to emulate a more realistic scenario.

## 5 Conclusion

This study provides evidence that SAM activity can be an indicator for increased levels of effortful listening. Unlike other reactions of the autonomic nervous system (e.g., skin conductance, pupil diameter, etc., see Mackersie and Cones, 2011), an increased activity of the vestigial pinna-orienting system (Hackley, 2015) could be interpreted as an attempt to alter the shape of the pinna or ear canal. This manipulation could potentially influence stimulus related factors in models of listening effort, such as the transmission factors as described in Pichora-Fuller et al. (2016), or external/exogenous factors in Strauss et al. (2010) and Strauss and Francis (2017). While increased activity of auricular muscles in response to automatic and intentional attention can lead to visible movements of the pinna (Strauss et al., 2020), it is currently not known if they are strong enough to achieve an actual benefit. Especially in the current experimental setup, without any spatial separation between target and distractor, orienting the pinna would be futile, even though the neural circuits may still activate the auricular muscles and attempt to aid stream segregation. Additionally, because the PAM, which is the second largest auricular muscle, did not show increased activity during effortful listening, any potential pinna movement would be severely limited. Furthermore, as head movements were restricted and stimuli were not lateralized, the question arises if the SAM would still show increased activity if participants would be able to orient their head toward a sound source, as head movements would have an appreciable impact on perception and task difficulty. Furthermore, it should be noted that the direction of the head (or gaze) and the intended listening direction are fairly often separated in a real-life scenario. Conversely, the ability to separate sound sources without explicit head movements is an important ability in order to understand speech in noise (see the “cocktail-party effect,” Cherry, 1953), which could be aided by pinna movement.

Nevertheless, future studies should focus on exploring the auricular muscles in the context of the multi-dimensional concept of listening effort (e.g., Alhanbali et al., 2019; Shields et al., 2023), which was present in the current study when comparing the SAM results to the self-reported perceived listening effort ratings. In this context, focusing on the participants losing the target stream would be of interest, as this self-reported measure seemed to resemble the SAM more closely than other self-reported measured. Overall, the investigation of auricular muscles (as well as facial muscles, which share neural pathways), as markers of effortful listening is practically non-existent in the current literature, and their addition might shed more light onto the dimensions of listening effort, especially because the intended effect of pinna movements is fairly easy to interpret from an evolutionary point-of-view (Hackley, 2015).

The activity of auricular muscles as an objective correlate for effortful listening could be utilized as a novel tool, or rather, an addition to more established tools, in cognitive neuroscience. Furthermore, it could be useful in human-machine interaction by monitoring the state of the user, especially because placing sensors around the ear can be done in a very unobtrusive manner. Lastly, it could be worthwhile to explore auricular muscle activity to potentially be used as an objective metric to assess the effectiveness of hearing aid algorithms to reduce listening effort, as there is a clear physiological connection between the pinna and auditory perception.

## Data Availability

The raw data supporting the conclusions of this article will be made available by the authors, without undue reservation.
